# The Development and Survival but Not Function of Follicular B Cells Is Dependent on IL-7Rα Tyr449 Signaling

**DOI:** 10.1371/journal.pone.0088771

**Published:** 2014-02-13

**Authors:** Daniel T. Patton, Adam W. Plumb, Stephen A. Redpath, Lisa C. Osborne, Georgia Perona-Wright, Ninan Abraham

**Affiliations:** 1 Department of Microbiology and Immunology, University of British Columbia, Vancouver, Canada; 2 Department of Zoology, University of British Columbia, Vancouver, Canada; Maisonneuve-Rosemont Hospital, Canada

## Abstract

IL-7 is a critical cytokine for lymphocyte development. Recent work has highlighted critical roles for IL-7 signaling in mature T cell homeostasis and function, but its role in B cells is less well characterized. Using a knock-in mouse possessing a Tyr to Phe mutation at position 449 (IL-7Rα^449F/449F^ mice) within the cytoplasmic SH2-binding motif of IL-7Rα, we evaluated the role of IL-7Rα Y449 motif in spleen B cells. IL-7Rα^449F/449F^ mice had reduced numbers and increased death of follicular B cells compared to WT, but had significantly more follicular cells than IL-7Rα^−/−^. The death of IL-7Rα^449F/449F^ follicular cells was not due to a failure to respond to BAFF or lower levels of BAFF, a critical B cell survival factor. Marginal zone B cells were unaffected by the IL-7Rα^449F/449F^ mutation. Any role for TSLP was ruled out, as TSLPR^−/−^ mice had an identical B cell phenotype to wild-type mice. Bone marrow chimeras and the absence of IL-7Rα on B cells suggested that IL-7 did not directly regulate mature B cells, but that an IL-7-responsive cell was influencing B cells. IL-7 was also critical at the checkpoint between the T1 and T2 stages in the spleen. IL-7Rα^−/−^ mice fail to develop T2 cells, but IL-7Rα^449F/449F^ show a reduction compared to WT but not complete absence of T2 cells. We also tested the functional responses of IL-7Rα^449F/449F^ to antigens and infection and found no difference in antibody responses to T-dependent or T-independent antigens, or to Influenza/A. IL-7 was important for generation of antibody responses to the intestinal worm *H. polygyrus* and for naive levels of IgA. Taken together, this suggests that IL-7 regulates follicular B cell numbers and survival in a cell-extrinsic manner, via a bone-marrow derived cell, but is not critical for antibody production outside the gut.

## Introduction

B cells are essential for the generation of antibody responses to pathogens. IL-7Rα detects two key cytokines, interleukin-7 (IL-7) and thymic stromal lymphopoietin (TSLP), which have been previously shown to regulate B cell development. IL-7Rα^−/−^ mice possess very few mature T or B cells, which has limited the analysis of the role of IL-7Rα in periphery. Here, we present work using mutant mice to analyze the role of IL-7Rα in peripheral B cell function and homeostasis.

Two main B cell lineages are found in the peripheral immune system, B1 and B2 B cells [1]. B2 cells are found in secondary lymphoid organs [2] and are further divided in the spleen by their anatomical location and phenotype. Follicular (FO) B cells exist in the follicular regions of the spleen, respond to T-dependent antigens and form germinal centers for the production of high-affinity antibody. Marginal zone (MZ) B cells are found in the regions surrounding the follicles, respond to T-independent type II antigens and rarely form germinal centers [3].

IL-7 is detected by the IL-7Rα-γc complex, whereas TSLP is detected by IL-7Rα-TSLPR. Despite the fact neither IL-7Rα nor TSLPR are expressed on peripheral resting B cells, generation of B2 lineages is dependent on IL-7, as in the absence of IL-7 or IL-7Rα signals, few follicular or marginal zone cells develop [4], [5]. The development of the remaining cells may be dependent on Flt3-L or TSLP[6], [7]. The remaining B2 cells in IL-7Rα^−/−^ and IL-7^−/−^ mice have a marginal zone phenotype but are not able to respond to T-independent type II immunization [8]. The role of IL-7 and IL-7Rα in the generation of B1 cells is still unclear; IL-7Rα^−/−^ mice have been reported to lack B1 cells [4], whereas IL-7^−/−^ do not [5], potentially leaving a role for TSLP. Over-expression of IL-7 [9] or TSLP [10] has been previously shown to result in expansion of the follicular B cell population.

Three conserved tyrosines in the cytoplasmic domain of IL-7Rα are found in all mammals. Tyr449 is part of an YVTM signaling motif, which is thought to bind STAT5 and the regulatory subunits of class IA PI3K. We previously generated IL-7Rα^449F/449F^ mice [11], which possess a point mutation that blocks signaling through the Tyr449 motif. We have shown that the IL-7Rα^449F/449F^ mutation causes loss of phosphorylation of STAT5 in T and early B cells [11], [12], as well as blocked development of T cells in the thymus and homeostasis in peripheral organs [11], [13]. The role of IL-7Rα Tyr449 has previously been investigated using chimeric receptors in bone marrow B cell culture, but this has not been assessed *in vivo*, nor with an intact receptor [14].

Although not the focus of this study, we have previously examined bone marrow B cell development in IL-7Rα^449F/449F^ mice. IL-7Rα^449F/449F^ and IL-7Rα^−/−^ mice show reduced immature IgM^+^IgD^−^ bone marrow cells. Throughout development, IL-7Rα^449F/449F^ and IL-7Rα^−/−^ mice have a similar phenotype, suggesting that Tyr449 is the key signaling residue for the development of bone marrow B cells.

IL-7 is critical for the generation of B cells in the bone marrow, but its role in the development and function of peripheral B cells is less clear, in part due to the severe phenotype seen in IL-7^−/−^ and IL-7Rα^−/−^ mice. The IL-7Rα^449F/449F^ mouse permits analysis of the function of IL-7 signaling without completely removing the receptor. Here, we investigate the role of the IL-7Rα Tyr449 motif in the development, homeostasis and function of peripheral B cells. We show that follicular B cell development and survival are regulated in a cell-extrinsic manner dependent on IL-7Rα Tyr449 signaling. However, primary antibody responses are not defective to the majority of antigens, with the exception of the response to *H. polygyrus* in the gut.

## Materials and Methods

### Mice

All mice were maintained in the Centre for Disease Modeling at UBC with full ethical and procedural approval from the University of British Columbia Animal Care and Biosafety Committee (Protocols A07-0115, A12-0118 and A12-0119). All work was carried out according to institutional guidelines. All efforts were made to minimize suffering, with minimally invasive procedures. IL-7Rα^449F/449F^ mice were previously generated by introducing a point mutation into the endogenous IL-7Rα gene and were backcrossed to C57BL/6 for 15 generations. All mice were bred on site and maintained under identical conditions. TSLPR^−/−^ mice were obtained from Dr James Ihle, transgenic IL-7 [15] and IL-7Rα^−/−^ (B6.129S7-*Il7r^tm1Imx^*/J), BoyJ (B6.SJL-*Ptprc^a^ Pepc^b^*/BoyJ), Rag1^−/−^ (B6.129S7-*Rag1^tm1Mom^*/J) were purchased from Jackson Laboratories.

### Flow cytometry

2-10×10^6^ cells were stained for 30 minutes on ice with antibodies detailed in Table S1. For intracellular staining, surface-stained cells were fixed and permeabilized using Foxp3 fix/perm kit (eBioscience) for 16 hours and then stained with intracellular antibodies. Samples were acquired on a LSRII or FACSCanto with 405 nm, 488 nm, 561 nm and 633 nm lasers. For flow-sorting experiments, cells were labeled with antibody cocktails, in some cases depleted of FITC-conjugated cells using anti-FITC beads and an AutoMacs (Miltenyi Biotech) and then flow-sorted using either FacsAria IIu or Influx machines (Becton-Dickinson). Analysis was carried out using Flowjo (Treestar, Ashland, Oregon), Excel and Prism.

### Caspase 3/7 activation assessment

Single-cell preparations of spleen cells were incubated for 30 mins at 37°C with 1∶10 dilution of CellEvent Caspase-3/7 reagent (Invitrogen). Cells were then washed, placed on ice and labeled with antibodies as above.

### Bone-Marrow Chimeras

Rag1^−/−^BoyJ mice (CD45.1^+^) were irradiated with two doses of 6.5 gy four hours apart. Twenty-four hours after irradiation, mice were injected via the tail vein with 5×10^6^ total bone marrow cells. Mice were then maintained on 2 mgml^−1^ Neomycin for two weeks after irradiation. The mice were then left for six to eight weeks to reconstitute, and then analyzed.

### Real-time PCR

Cells were placed into Trizol (Invitrogen) and RNA purified as specified previously. Complementary DNA was then made from total RNA using Superscript III (Invitrogen) or Maxima (Thermo) cDNA synthesis kits. Real-time PCR was then performed using Ssofast EvaGreen mastermix (Biorad), CFX96 PCR machine. Primers used: Rps29-Fwd: ACGGTCTGATCCGCAAATAC Rps29-Rev CATGATCGGTTCCACTTGGT; Dtx1-Fwd GTGCCCTACATCATCGACCT Dtx1-Rev CCGACGATGGATCGTAGAAG Dtx2 Fwd GACTCAGTTTCGCCAGAACA Dtx2-Rev GCTACCCAGGATCCGTCAT. Primers for Notch2, Jag1, Jag2 and Dll1 were taken from Gazit *et al* [16]. Cycling conditions were: 95°C 2 mins followed by 40 cycles of 95°C for 10 sec and 60°C for 15 sec. Finally, a melt curve was performed to check for presence of a single product from each reaction. Expression level of genes-of-interest were then compared to expression of ribosomal protein *RPS29.*

### Infection and Immunization

Mice were immunized with either 100 µgml^−1^ NP-KLH absorbed onto alum i.p., 50 µgml^−1^ NP-LPS i.p. (both Biosearch Technologies). For influenza experiments, mice were infected with with 5 HAU of Influenza A PR8 i.n. as described previously[17]. Mice were inoculated by gavage with 200 *Heligmosomoides polygyrus bakeri* infective third-stage larvae, as described [18], [19]. Mice were euthanized after 14 days (NP-KLH, NP-LPS or Influenza) or 21 days (*H. polygyrus*) and blood taken by cardiac puncture. BAL was obtained by flushing the lungs of mice with 1 ml PBS.

### Antibody Levels

Antibody levels in sera and BAL were detected by ELISA. Briefly, plates were coated with antigen or anti-mouse pan-Ig antibody and then serial dilutions of serum or BAL fluid added to the plates. The bound antibody was then detected using a SBA Clonotyping System-B6/C57J-HRP kit (Southern Biotech) and ABTS reagent (Sigma-Aldrich). We then calculated the dilution of serum or BAL which gave 50% of the maximal responses using GraphPad.

Flu-specific antibodies were coating a plate with 500 HAU ml^−1^ heat-killed Influenza A PR8; NP-specific responses were detected using NP-BSA (Biosearch) coated plates. HES-specific responses were detected by coating a plate with 1 µgml^−1^ HES (a kind gift of Rick Maizels, Edinburgh, UK).

### BAFF serum levels

BAFF serum levels were assessed using the Mouse BAFF/BLyS/TNFSF13B Quantikine ELISA Kit (R&D Systems), and concentrations obtained by comparing to a standard curve.

### Microscopy

Spleens were embedded in OCT, frozen and 7 µm sections were taken. Sections were then stained with anti-B220, anti-IgM and anti-IgD, and analyzed on an Olympus Fluoview FV1000 microscope.

## Results

### IL-7 controls the number of follicular B cells in the spleen

**Figure 1:**
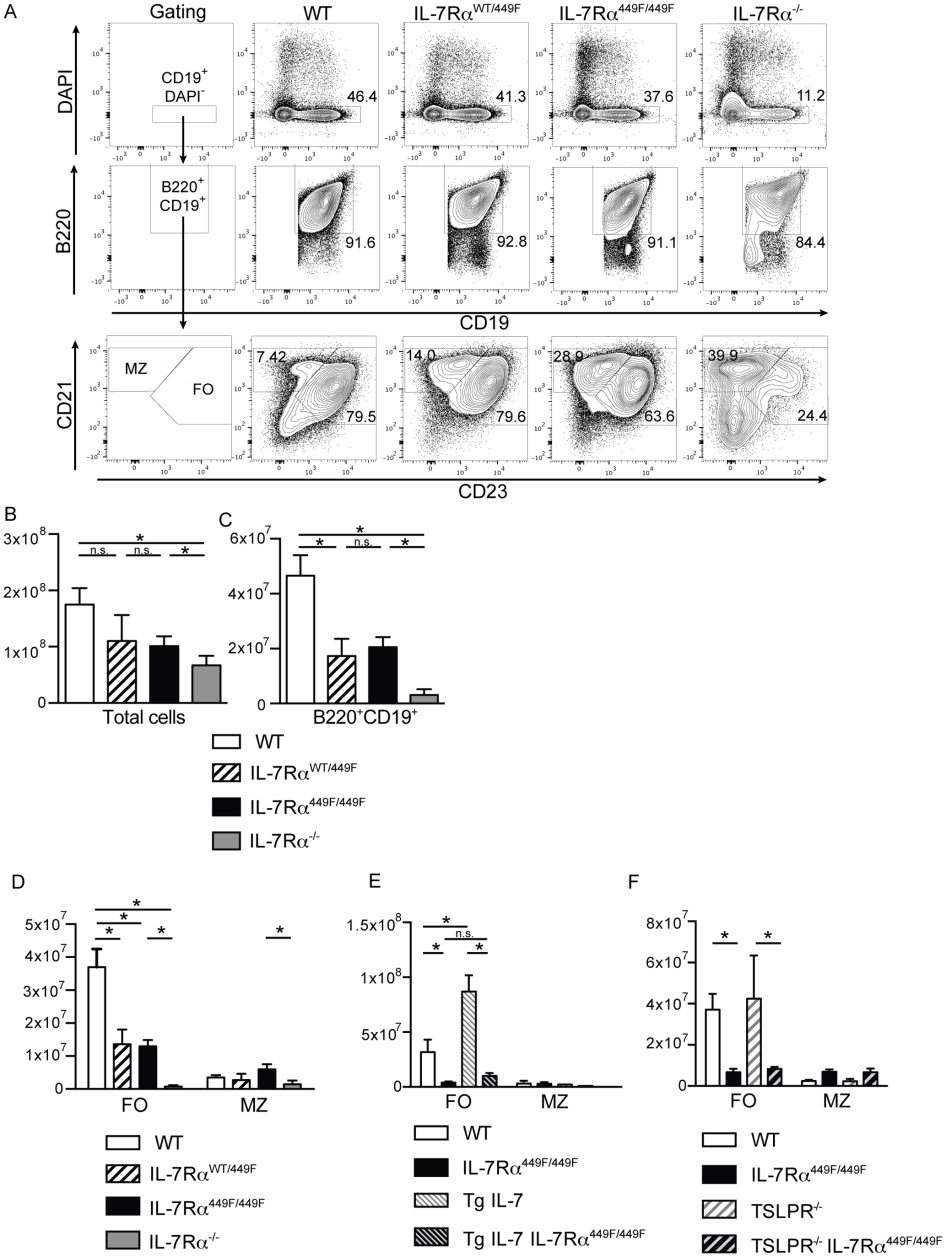
IL-7, not TSLP, controls the number of follicular B cells in the spleen. (A) Spleens from WT, IL-7Rα^WT/449F^, IL-7Rα^449F/449F^ and IL-7Rα^−/−^ mice were taken and stained with antibodies directed against CD19, B220, CD23 and CD21. (B) Total numbers of spleen cells and (C) Numbers of B cells from mice shown in A. Numbers of follicular (CD19^+^B220^+^CD21^+^CD23^+^) and marginal zone (CD19^+^B220^+^CD21^hi^CD23^lo^) from (D) WT (n = 4), IL-7Rα^WT/449F^ (n = 3), IL-7Rα^449F/449F^ (n = 5) and IL-7Rα^−/−^ (n = 4) (E) WT (n = 3), IL-7Rα^449F/449F^ (n = 3) and IL-7 over-expressing (TgIL-7) (n = 3) and Tg IL-7 IL-7Rα^449F/449F^ (n = 3). (F) WT (n = 4), IL-7Rα^449F/449F^ (n = 4), TSLPR^−/−^ (n = 4) and TSLPR^−/−^ IL-7Rα^449F/449F^ (n = 6) mice.

To examine the role of IL-7 signaling in generating peripheral B cells, we decided to examine splenic B cells under conditions of limited IL-7Rα signaling. Total numbers of splenocytes as well as live B cells, based on their expression of B220 and CD19, were reduced in IL-7Rα^449F/449F^ and IL-7Rα^−/−^ mice, suggesting that IL-7Rα signaling plays a critical role in the development of peripheral B cells (Figure 1A, 1B). Heterozygous IL-7Rα^WT/449F^ had similar numbers of B cells to IL-7Rα^449F/449F^ mice, suggesting that lacking a single functional copy of IL-7Rα Tyr449 prevents normal B cell numbers. We then examined the two main B2 subsets in the spleen, follicular and marginal zone B cells, given the previously reported differences in FO and MZ cells numbers in mice which overexpress IL-7 [9]. We confirmed that IL-7Rα^−/−^ mice had severely reduced follicular and marginal zone cells; the small number of cells which remained in these mice mostly possessed a marginal zone phenotype (CD21^hi^CD23^lo^), although there were smaller populations of follicular (CD23^+^CD21^+^) and CD23^−^CD21^−^ cells. However, unlike IL-7Rα^−/−^, IL-7Rα^449F/449F^ mice had normal numbers of marginal zone cells, but reduced follicular B cells compared to WT, suggesting that signals downstream of the IL-7Rα Tyr449 influenced the numbers of follicular B cells but were dispensable for marginal zone B cells (Figure 1A, 1C).

We then tested if increasing the level of IL-7Rα signaling by over-expression of IL-7 results resulted in more follicular B cells. We examined B cell populations in IL-7-overexpressing transgenic mice (Tg IL-7) and, confirming previous observations [9], found increased numbers of follicular and similar levels of marginal zone B cells to wild-type mice. To then examine if increasing IL-7 can rescue the phenotype of the IL-7Rα^449F/449F^ mice, we crossed them to generate IL-7Rα^449F/449F^ Tg IL-7 mice. Transgenic over-expression of IL-7 could not rescue the phenotype of reduced follicular cells seen in IL-7Rα^449F/449F^ mice, which suggested that Tyr449 was required for the maintenance of follicular B cell numbers (Figure 1D).

As IL-7Rα can bind both IL-7 and TSLP, we used TSLPR^−/−^ mice to examine which was the key cytokine for follicular B cell numbers. TSLPR^−/−^ showed similar follicular and marginal zone levels to WT mice, suggesting that TSLP did not play a role in this process (Figure 1E). IL-7Rα^449F/449F^ TSLPR^−/−^ also did not show any difference in the number of follicular nor marginal zone B cells compared to IL-7Rα^449F/449F^, suggesting that even in the absence of normal IL-7 signals, TSLP cannot compensate (Figure 1F). This suggests that IL-7 was the key cytokine for follicular B cell numbers, not TLSP.

These experiments suggested that IL-7 had control of the number of follicular B cells in mice. Increasing IL-7 levels resulted in increased numbers of follicular cells while reduced IL-7Rα signaling resulted in fewer follicular B cells. However, IL-7 Tyr449 dependent signaling was not required for marginal zone B cells.

### MZ and FO B cells are in correct physiological locations and are phenotypically normal similar in IL-7Rα^449F^ mice

**Figure 2:**
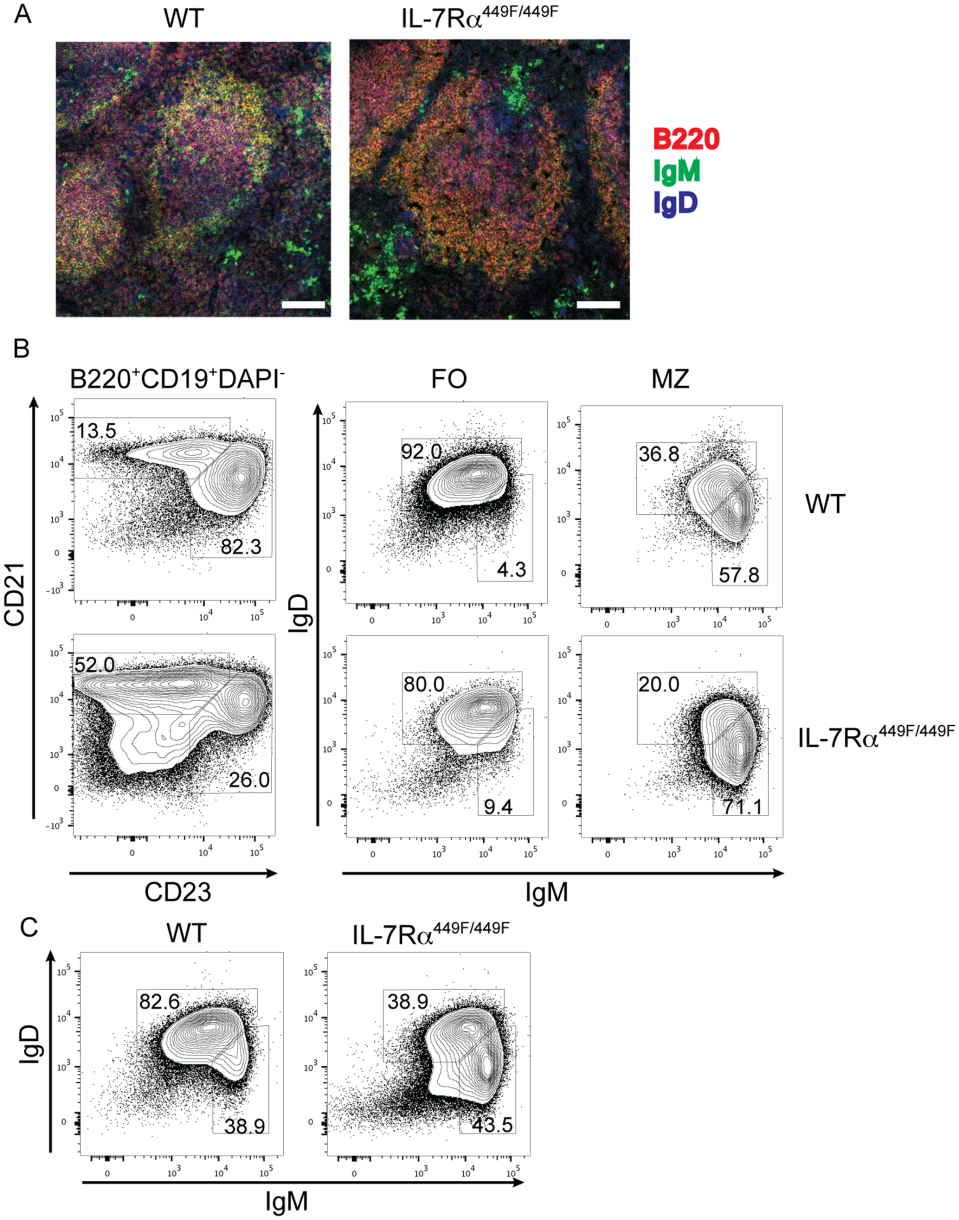
FO and MZ cells in IL-7Rα^449F^ exist in correct physiological locations and express normal levels of IgM and IgD. (A) Sections of spleens from WT and IL-7Rα^449F^ mice were taken and stained with antibodies to IgD, IgM and B220 to reveal follicular and marginal zone cells. Scale bar is 100 µm (B) Marginal zone and follicular B cells from WT and IL-7Rα^449F^ mice were stained with antibodies to IgM and IgD. (C) Shows complete CD19^+^B220^+^ population from (B).

To determine if the cells were in the correct physiological locations, we examined the organization of the spleen. WT mice showed a large IgD^+^B220^+^ follicular B cell compartment and a small layer of B220^+^IgM^+^ marginal zone B cells outside that. IL-7Rα^449F/449F^ spleens showed a thicker marginal zone compartment relative to the follicle, confirming the observations seen by flow cytometry (Figure 2A). We also examined expression of IgM and IgD by FO and MZ cells in WT and IL-7Rα^449F^ mice. Both marginal zone and follicular B cells show normal expression of IgM and IgD. Within the B220^+^ population, we also see a reduction in IgD^+^IgM^low^ follicular cells, and an increase in IgM^hi^IgD^low^ marginal zone cells in IL-7Rα^449F^, similar to that seen by CD21-CD23 staining (Figure 2C, compare to 1A).

### IL-7 is required in non-B cells for follicular B cell homeostasis

The reduction seen in follicular B cells could be a result of either cell-intrinsic IL-7Rα within the B cells or due to IL-7Rα signaling within other cells influencing the B cell population. To examine this, we used mixed bone-marrow chimeras to examine the spleen defect seen in IL-7Rα^449F/449F^ mice. We transferred WT or IL-7Rα^449F/449F^ bone marrow to RAG1^−/−^ mice to determine if the FO/MZ phenotype found in the mice was dependent on hematopoietic cells or due to defects in splenic architecture or IL-7Rα signaling in non-bone marrow derived cells. We also injected lethally irradiated RAG1^−/−^ mice with a mixture of CD45.1^+^ BoyJ and CD45.2^+^ WT or IL-7Rα^449F/449F^ bone marrow in a 1∶9 ratio. We used a 1∶9 ratio as this gave a sufficiently large population of spleen B cells in the IL-7Rα^449F/449F^ mice, as IL-7Rα^449F/449F^ precursor B cells were out-competed by WT cells in the bone marrow (Unpublished data).

**Figure 3:**
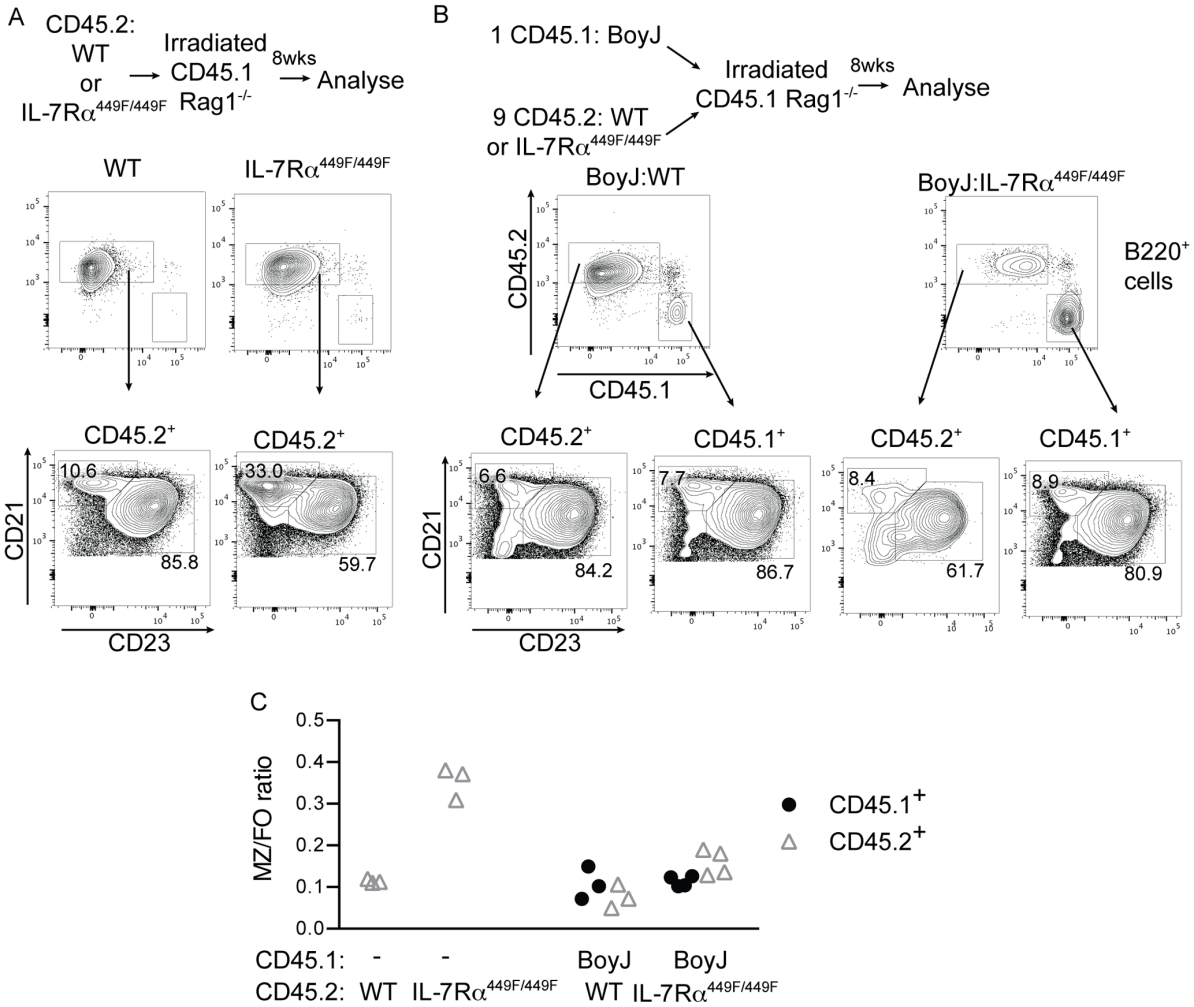
Presence of WT cells rescues IL-7Rα^449F/449F^ FO-MZ phenotype. Irradiated Rag1^−/−^ mice were injected with (A) WT (n = 3) or IL-7Rα^449F/449F^ (n = 3) bone marrow alone or (B) a 9∶1 mixture of WT (n = 3) or IL-7Rα^449F/449F^ (n = 4) to B6.SJL bone marrow or and left for eight weeks to reconstitute. The proportion of CD45.1^+^ (BoyJ) and CD45.2^+^ (either WT or IL-7Rα^449F/449F^) B220^+^CD21^+^ cells are shown, with the FO (CD21^+^CD23^+^) and MZ (CD21^hi^CD23^lo^) populations. The MZ/FO ratio is then shown (C) from the CD45.1^+^ and CD45.2^+^ populations. Experiments were repeated with at least three separate sets of chimeras, with similar results.

WT bone marrow transferred to RAG1^−/−^ mice fully reconstituted the spleen and showed similar B cell follicular-marginal zone profile to WT mice (Figure 3A and 3C, compare to 1A). Mice which received IL-7Rα^449F/449F^ bone marrow showed a phenotype similar to that seen in intact IL-7Rα^449F/449F^ mice, with a relatively larger marginal zone and smaller follicular population (compare 2B and 1A). This suggests that the reduction in follicular cells in IL-7Rα^449F/449F^ mice is due to defects within the bone marrow derived cells, not aberrant development of spleen architecture or IL-7Rα signaling within non-hematopoietic cells.

We then examined mixed WT:BoyJ and IL-7Rα^449F/449F^:BoyJ chimeras. As expected, WT and BoyJ cells showed identical follicular and marginal zone proportions in the CD45.1^+^ (BoyJ) and CD45.2^+^ (WT) populations from the same mouse (Figure 3B and 3C). Remarkably, IL-7Rα^449F/449F^ CD45.2^+^ cells show an identical FO-MZ ratio to BoyJ CD45.1^+^ cells from the same mouse. This data suggests that the lack of follicular cells in IL-7Rα^449F/449F^ mice is due to an environmental factor outside the B cells, and that the defect seen in IL-7Rα^449F/449F^ mice was rescued by the presence of WT cells. IL-7Rα^449F/449F^ cells within the WT: IL-7Rα^449F/449F^ chimeras still had defective bone marrow B cell development, which resulted in fewer cells arriving in the spleen, but normal spleen development into FO and marginal zone cells. We could also not find expression of IL-7Rα on any B220^+^CD19^+^ cells from either WT or IL-7Rα^449F/449F^ mice (not shown), which again suggests that the effects of IL-7 on B cells in the spleen were cell-extrinsic.

**Figure 4:**
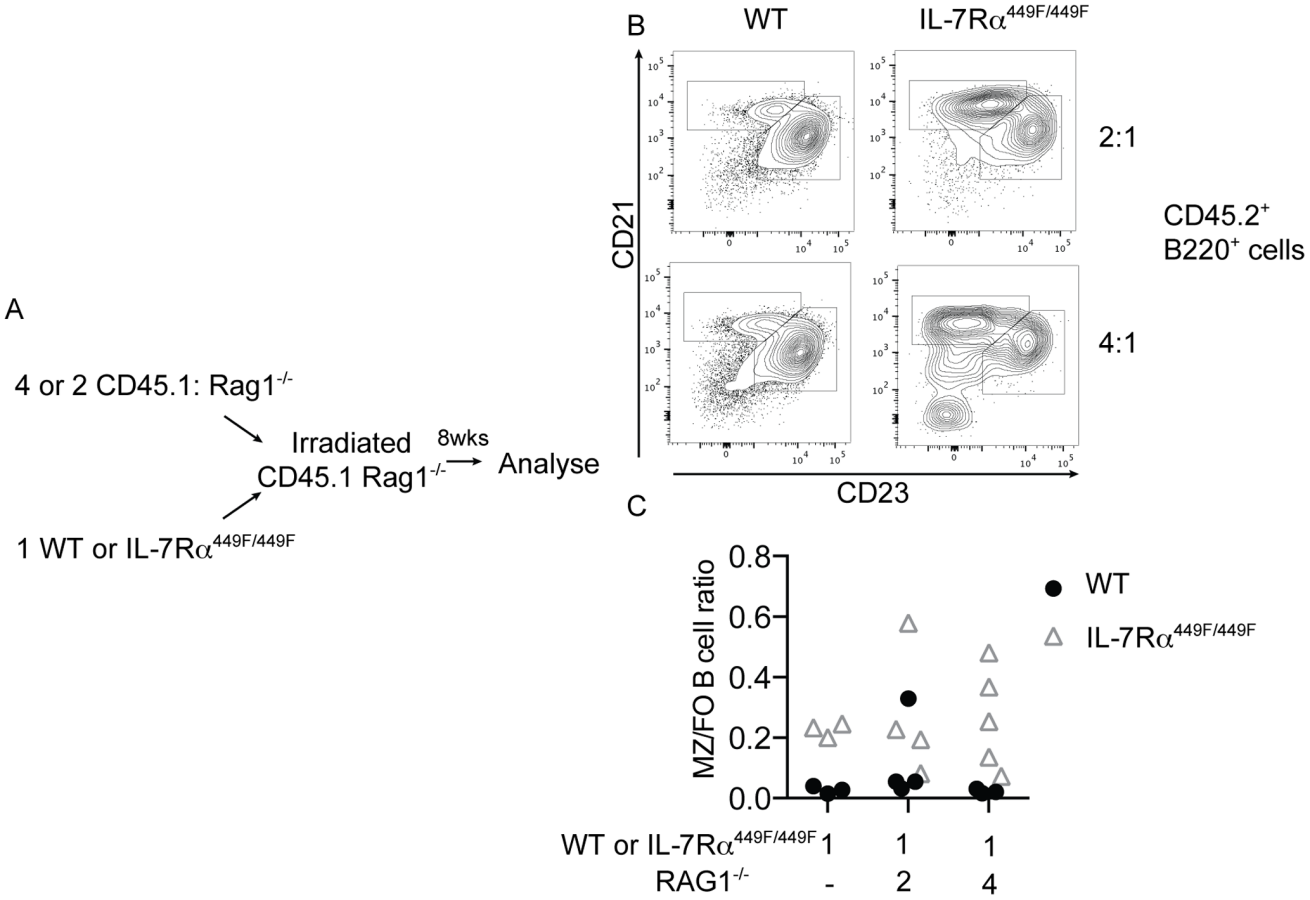
Follicular B cells levels in IL-7Rα^449F/449F^ mice are dependent on a RAG-dependent non-B cell. (A) Irradiated Rag1^−/−^ mice were injected with a mixture of CD45.2^+^ WT or IL-7Rα^449F/449F^ and CD45.1^+^Rag1^−/−^ bone marrow at a ratio of 1∶2 or 1∶4. Mice injected with only WT or IL-7Rα^449F/449F^ bone marrow were also generated in parallel. (B) Shown are the MZ and FO populations from B220^+^CD45.2^+^ cells from these chimeras after eight weeks reconstitution. The MZ to FO ratios are shown in (C), with each symbol indicating an individual mouse.

The mixed BoyJ:IL-7Rα^449F/449F^ chimeras described above suggested that the presence of WT bone marrow derived cells could rescue the follicular B cell phenotype seen in IL-7Rα^449F/449F^ mice. To further narrow the cell types which could influence the follicular B cell numbers, we injected lethally irradiated CD45.1^+^RAG1^−/−^ mice with a mixture of CD45.1^+^ RAG1^−/−^ and either CD45.2^+^ WT or IL-7Rα^449F/449F^ bone marrow (Figure 4A). RAG1^−/−^ bone marrow was given in excess at a ratio of either 2 or 4 RAG1^−/−^ cells to 1 WT or to IL-7Rα^449F/449F^ cell, to provide a non-RAG1 dependent immune system that had the greatest proportion of WT cells possible. This generated mice which possess an immune system which is predominantly WT in the non-RAG-dependent compartments (granulocytes, DC, NK etc.), but either WT or IL-7Rα^449F/449F^ in the RAG-dependent (T and B) cell compartment.

Mice which received WT and RAG1^−/−^ bone marrow, as expected, showed a similar B cell FO/MZ ratio to mice which received WT bone marrow only (Figure 4B, compare to 3B). Mice which received a mixture of IL-7Rα^449F/449F^ and RAG1^−/−^ bone marrow showed similar proportions of follicular B cells and a similar marginal zone-follicular ratio to mice which had received only IL-7Rα^449F/449F^ bone marrow (Figure 4B and 4C, compare to 3B). This is in contrast to the experiments in 3A and 3B, where WT T and B cells were present and the IL-7Rα^449F/449F^ B cell population showed a similar phenotype to the WT. These experiments, taken together, suggest that a bone marrow derived, RAG1-dependent cell is influencing follicular B cell numbers.

### IL-7Rα mutant mice have fewer T2 stage cells in the spleen

**Figure 5:**
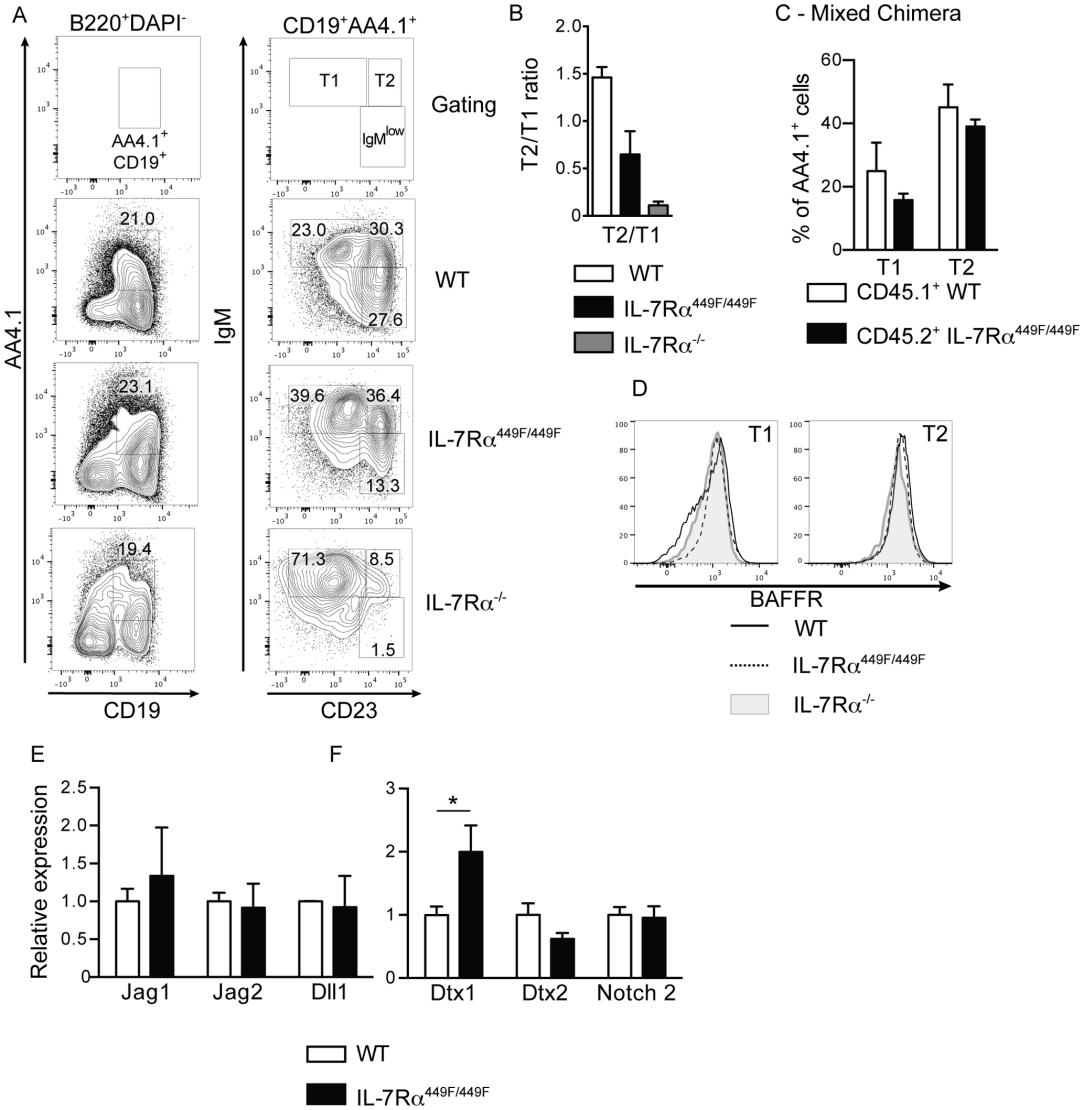
IL-7Rα^449F/449F^ and IL-7Rα^−/−^ B cells are arrested at the T1 stage within the spleen. (A) Expression of CD19 and AA4.1 on B220^+^DAPI^−^ cells in the spleen, and the proportions of T1 (IgM^+^CD23^−^) and T2 cells (IgM^+^CD23^+^) cells within the AA4.1^+^ population. (B) Ratio of T2 cells to T1 in the spleens of WT (n = 4), IL-7Rα^449F/449F^ (n = 3) and IL-7Rα^−/−^ mice (n = 5). (C) Proportions of T1 and T2 populations in 9∶1 BoyJ: IL-7Rα^449F/449F^ mixed bone marrow chimeras (n = 4) from figure 2B. (D) Surface expression of BAFFR on T1 and T2 cells from WT, IL-7Rα^449F/449F^ and IL-7Rα^−/−^ mice. (E) RNA levels of notch ligands Jag1, Jag2 and Dll2 in the spleen of WT, IL-7Rα^449F/449F^ and IL-7Rα^−/−^ mice. (F) Expression of Deltex1, Deltex2 and Notch2 on sorted spleen T1 cells. Shown is mean of three cDNA preparations for each genotype.

B cells undergo a series of developmental stages in the spleen before they become follicular or marginal zone B cells. We therefore examined if B cells in the spleens of IL-7Rα^449F/449F^ and IL-7Rα^−/−^ mice were failing to progress before reaching the follicular and marginal zone stages. B cells arrive in the spleen from the bone marrow as IgM^+^AA4.1^+^ immature B cells, where they can be divided based on the expression of CD23 into T1 (IgM^+^CD23^low^) and T2 (IgM^+^CD23^+^) cells. Marginal zone and follicular B cells are thought to differentiate from the T2 cells [20]. IL-7Rα^449F/449F^ and IL-7Rα^−/−^ mice had increased proportion of T1 cells (B220^+^CD19^+^AA4.1^+^IgM^+^CD23^low^; Figure 5A). However, IL-7Rα^−/−^ had a drastic reduction in T2 cells; IL-7Rα^449F/449F^ mice showed a reduction in ratio of T2 to T1 cells when compared to WT, suggesting a clear T1-T2 defect in IL-7Rα^449F/449F^ and IL-7Rα^−/−^ mice (Figure 5B). This shows that there are IL-7Rα Y449-independent signaling events involved in lymphopoiesis.

We also examined T1 and T2 cells in the mixed bone marrow chimeras described in figure 3B. Development of T1 and T2 cells was identical in WT and IL-7Rα^449F/449F^ cells from the same mouse, suggesting cell-extrinsic factors regulated the development of cells at this stage (Figure 5C).

Two major pathways govern the development and survival of T1 and T2 cells in the spleen, Notch and BAFF [20]. BAFFR levels on the B cells were similar on T1 and T2 cells from WT, IL-7Rα^449F/449F^ and IL-7Rα^−/−^ mice (Figure 5D). Levels of RNA encoding Notch ligands Jagged1, Jagged2 and Delta-like-ligand 1 were equal between WT and IL-7Rα^449F/449F^ spleens (Figure 5E); however, levels of the negative regulator of notch signaling Deltex1 were notably higher in T1 cells from IL-7Rα^449F/449F^ mice (Figure 5F). This suggests that T1 cells may have altered Notch signaling in IL-7Rα^449F/449F^ mice, affecting the generation of T2 cells.

### IL-7 signals control follicular, but not marginal zone, B cell survival

**Figure 6:**
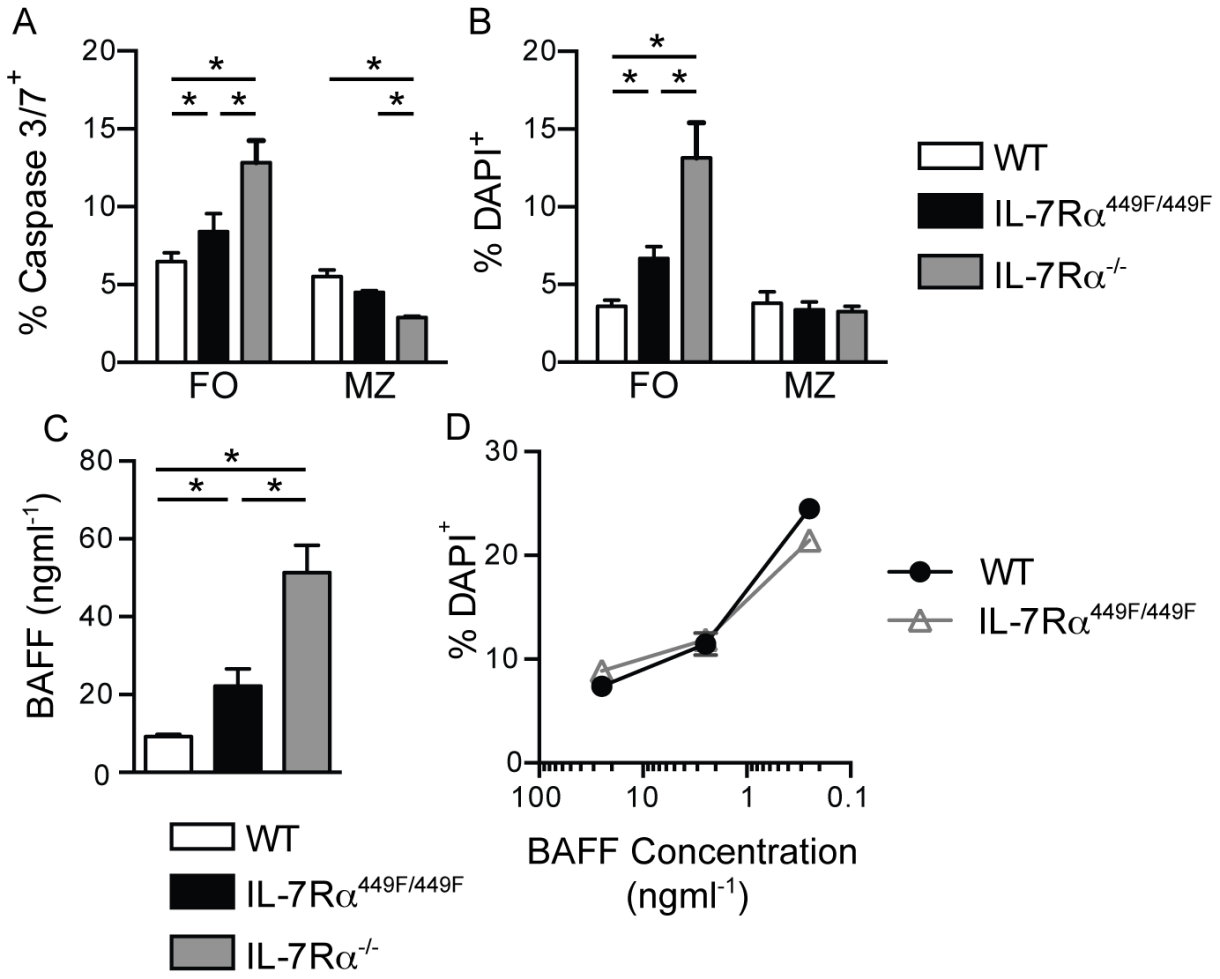
IL-7Rα signaling controls follicular B cell survival, but not BAFF signaling or responsiveness. Spleens from WT (n = 4), IL-7Rα^449F/449F^ (n = 5) and IL-7Rα^−/−^ (n = 4) mice were taken and either stained with DAPI immediately before acquisition (A) or Caspase3/7 dye for 30 minutes at 37°C (B), and then analyzed by flow cytometry. Shown is percentage positive for DAPI or Caspase 3/7 activation, and is representative of three separate experiments. (C) Levels of BAFF in serum from WT (n = 4), IL-7Rα^449F/449F^ (n = 3) and IL-7Rα^−/−^ mice (n = 3). Two separate sets of serum were analyzed. (D) Live follicular B cells from WT and IL-7Rα^449F/449F^ spleens were cultured for three days in BAFF. Shown is mean proportion of dead (DAPI^+^) cells assessed by flow cytometry after three days. Culture experiments were repeated three times.

To evaluate developmental progress beyond the T2 stage for the basis of the difference in follicular B cell versus marginal zone cells in IL-7Rα^449F/449F^ mice, we examined cell survival. The proportion of apoptotic (active Caspase3/7^+^) and dead (DAPI^+^) cells were examined in WT, IL-7Rα^449F/449F^ and IL-7Rα^−/−^ mice. In both IL-7Rα^449F/449F^ and IL-7Rα^−/−^ mice, dead and apoptotic cells were increased in follicular, but not marginal zone B cells (Figure 6A and 6B).

The major B cell survival factor is BAFF [21]. Mice treated with an anti-BAFF-R antibody show decreases in T2 cells and follicular B cells in the spleen [22], similar to IL-7Rα^449F/449F^ mice. We therefore decided to study the BAFF levels and BAFFR signaling in the WT, IL-7Rα^449F/449F^ and IL-7Rα^−/−^ mice. We found increased levels of BAFF in the serum of IL-7Rα^449F/449F^ and even higher amounts in IL-7Rα^−/−^ mice (Figure 6C), suggesting that defect in follicular B cells was not due to absence of BAFF. However, it remains a possibility that in the presence of adequate levels of BAFF, IL-7Rα^449F^ or KO cells were not responsive to BAFF. To test this hypothesis, we then examined BAFF-reactivity of follicular cells from WT and IL-7Rα^449F/449F^ mice. Spleen cells were centrifuged on Lympholyte-M to obtain live cells, and then sorted for follicular cells. The cells were then incubated for three days in BAFF, and then analyzed by flow cytometry for dead DAPI^+^ cells (Figure 6D). Unlike *ex vivo* cells (Figure 6B), cultured IL-7Rα^449F/449F^ follicular B cells have no defect in survival compared to WT, and respond equally well to BAFF. This suggested that IL-7Rα^449F/449F^ cells were able to respond normally to BAFF, but they do not receive adequate survival signals *in vivo* via a cytokine other than BAFF.

### IL-7Rα is required for IgA production, but not responses to immunization or pathogens

**Figure 7:**
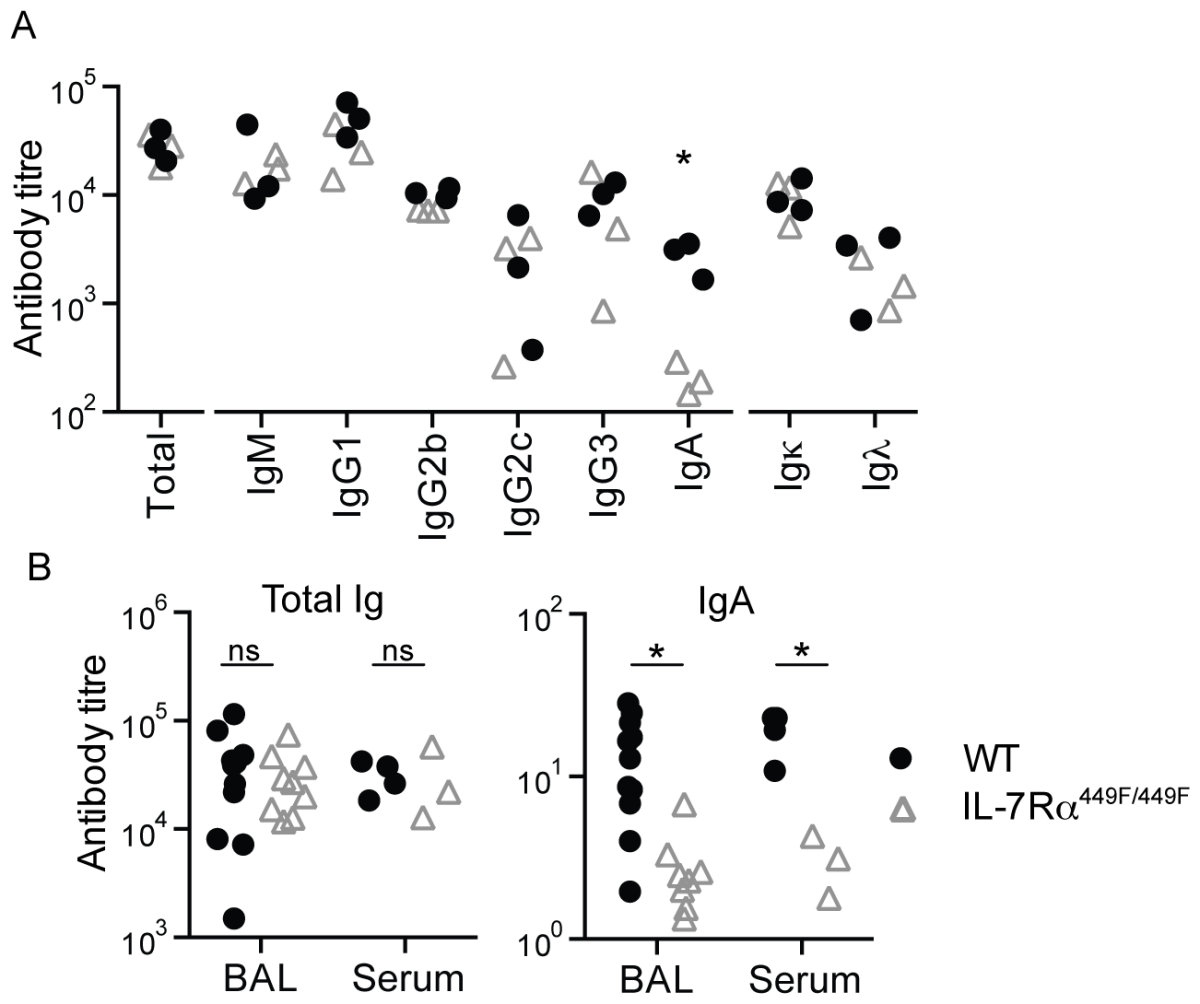
IL-7Rα^449F/449F^ mice have defective IgA production. (A) Resting antibody levels in serum from ten-week-old WT and IL-7Rα^449F/449F^ mice. (B) Comparison of total Ig and IgA levels in serum and broncho-alveiolar lavage fluid (BAL) taken from the same mice, with each symbol indicating an individual mouse.

Having shown a defect in the number of follicular B cells in the spleen of IL-7Rα^449F/449F^ mice, we then examined if the B cells from these mice were able to produce antibodies in the same levels as WT. Resting levels of circulating antibodies in sera taken from WT and IL-7Rα^449F/449F^ mice were assessed by ELISA. We found that the level of all antibody isotypes were identical between WT and IL-7Rα^449F/449F^ mice, with the exception of IgA (Figure 7A). We then investigated if IgA levels were similarly decreased in the lung, a major site of IgA function. Similar to the serum, we also saw a clear decrease in IgA levels in washings from the lung (BAL; broncho-alveolar lavage) in IL-7Rα^449F/449F^ mice (Figure 7B). This suggested that resting levels of antibodies of most isotypes were intact in IL-7Rα^449F/449F^ mice, with the exception of IgA.

**Figure 8:**
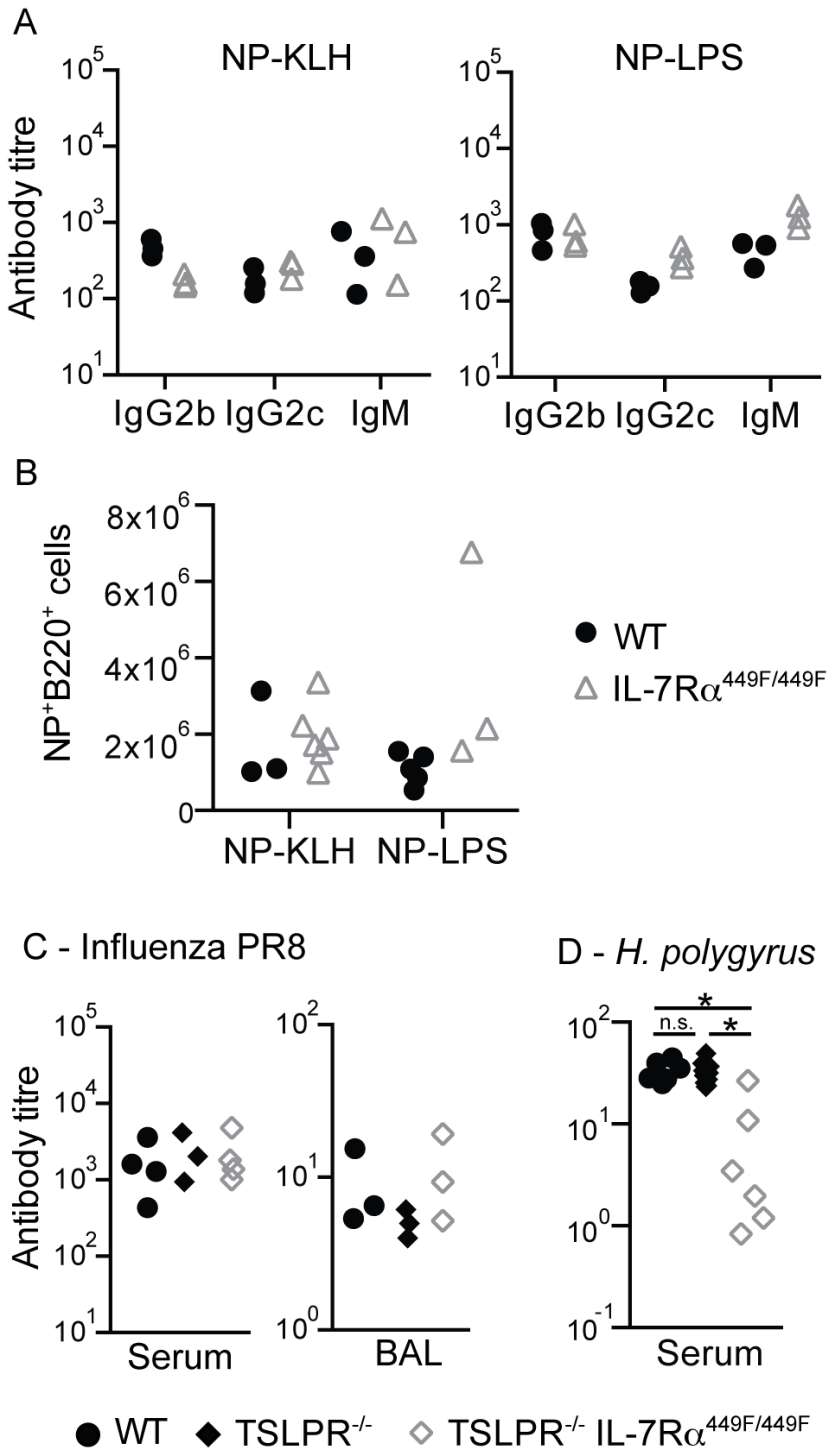
IL-7Rα^449F/449F^ is not required for primary antibody responses. (A) Mice were immunized with NP-KLH or NP-LPS to assess T-dependent and T-independent responses, respectively. NP-specific IgG2b, IgG2c and IgM antibody responses to T-dependent (NP-KLH) and T-independent antigens (NP-LPS) were assessed by ELISA. (B) Using a NP-PE fluorescent label, numbers of NP-specific B220^+^ cells were assessed in the spleen of WT and IL-7Rα^449F/449F^ mice. Two separate sets of mice were analyzed. (C) WT, TSLPR^−/−^ and TSLPR^−/−^IL-7Rα^449F/449F^ mice were infected with Influenza/A PR8 and strain-specific antibody responses in the serum and BAL were assessed by ELISA. n.d.  =  none detected. Three sets of mice were infected and analyzed. (D) WT, TSLPR^−/−^ and TSLPR^−/−^IL-7Rα^449F/449F^ mice were orally infected with *H. polygyrus* and antibody responses to secreted antigen in serum (HES) were assessed by ELISA at 21 days. n.d.  =  none detected. Each symbol indicates an individual mouse and is representative of two separate experiments.

To examine the B cell function of the IL-7Rα^449F/449F^ mice, we used the model antigens NP-KLH (T-dependent) and NP-LPS (T-independent type I). IL-7Rα^449F/449F^ mice were able to respond identically to WT to both antigens, as assessed by NP-specific IgG2b, IgG2c and IgM responses in serum and total numbers of NP-specific B cells in the spleen of these mice (Figure 8A and 8B). IgA responses in this assay were not detectable in WT, hence, we were unable to assess the role of IL-7R signaling in IgA producing B cells.

We then infected WT, TSLPR^−/−^ and TSLPR^−/−^ IL-7Rα^449F/449F^ mice with PR8 Influenza/A or the intestinal parasite *Heligmosomoides polygyrus* to assess the responses of B cells at mucosal sites, where IgA responses may be critical. We used TSLPR^−/−^ and TSLPR^−/−^ IL-7Rα^449F/449F^ mice to determine if the cytokine involved was TSLP or IL-7. In response to Influenza/A, the levels of PR8-specific IgG2b antibody produced by TSLPR^−/−^ and TSLPR^−/−^ IL-7Rα^449F/449F^ mice in the serum and BAL were identical to WT at day 14 after infection, suggesting that antibody responses to Influenza/A are not affected by the absence of either TLSPR or IL-7Rα Tyr449 dependent signaling (Figure 8C). In mice infected with *H. polygyrus*, WT and TSLPR^−/−^ mice produced robust anti-HES antibody responses, whereas TSLPR^−/−^ IL-7Rα^449F/449F^ mice showed reduced levels of anti-HES (Figure 8D). This suggest that the antibody response to *H. polygyrus*, but not Influenza/A, is dependent on IL-7 signaling through IL-7Rα Tyr449. We could detect neither PR8-specific nor HES-specific IgA in any of these samples, even in WT mice.

## Discussion

IL-7Rα signaling controls the number of follicular B cells in the spleen, but not their function. We have shown that reduction of IL-7Rα signaling, through mutation of the Y449 motif, reduced the number of follicular but not marginal zone B cells in the spleen. Increasing IL-7 has the opposite effect, increasing follicular B cells, but not marginal zone B cells. The critical cytokine was IL-7, not TSLP, as even in the absence of normal IL-7Rα signaling, TSLP did not compensate.

The reduction in follicular B cells found in IL-7Rα^449F/449F^ mice was not dependent on defective splenic architecture, as RAG1^−/−^ mice which had received IL-7Rα^449F/449F^ bone marrow show a similar phenotype to IL-7Rα^449F/449F^ mice. The mixed bone marrow chimera experiments also identified that IL-7 is not signaling in the B cells to influence their development, but instead in a RAG1-dependent non-B cell. The identity of this cell is unclear, but it is possibly a T cell, or a type-III ILC which could be influenced by the lack of lymphocytes. This conclusion is in keeping with the fact that B cells do not express IL-7Rα past the immature IgM^+^IgD^−^ stage in the bone marrow. We confirmed that IL-7Rα^449F/449F^ B cells do not have aberrant expression of IL-7Rα due to the generation of the knock-in mouse (not shown). Taken together, this information suggests that an IL-7Rα^+^ RAG-dependent cell was responsible for the homeostasis of the follicular B cell population, but how is still an open question. We ruled out the contribution of the key B cell survival cytokine BAFF, as levels of the cytokine were increased and *in vitro* BAFF responsiveness was normal in IL-7Rα^449F/449F^ follicular B cells. We speculate that the reason for enhanced BAFF levels in IL-7Rα^449F^ and IL-7Rα^−/−^ mice was due to reduced consumption of the cytokine due to fewer B cells.

The development of peripheral B cells in IL-7 mutant mice has been described previously, but these were cases where the peripheral development is severely perturbed especially in the complete absence of IL-7 signals, in the case of IL-7^−/−^ and IL-7Rα^−/−^ mice [4], [23]. Both have a preponderance of MZ-like cells that were unable to function normally when transferred to IL-7 replete mice [23].

Ceredig *et al* [9] showed that Tg IL-7 mice showed enhanced numbers of follicular cells, and this effect was not due to any defect in splenic architecture. This study also concluded that the enhanced production of B cell precursors in the bone marrow was responsible for the increases seen in follicular B cells. However, in our loss of function IL-7Rα^449F/449F^ mouse model, we would argue against reduced precursor B cell development being responsible for the changes in follicular B cells. Equal numbers of precursor T1 spleen B cells are found in IL-7Rα^449F/449F^ and IL-7Rα^−/−^ mice yet drastic differences are then seen in the FO and MZ compartments in these mice. If the differences in MZ and FO cells in IL-7Rα^449F/449F^ and IL-7Rα^−/−^ mice were due to enhanced BM lymphopoiesis in IL-7Rα^449F/449F^ compared to IL-7Rα^−/−^ mice, we would expect to see more T1 cells in IL-7Rα^449F/449F^ than in IL-7Rα^−/−^ mice, which we do not.

The difference between the B cell populations in IL-7Rα^449F/449F^ and IL-7Rα^−/−^ mice may be primarily in the failure of IL-7Rα^−/−^ B cells at the AA4.1^+^ transitional stage in the spleen. T2 cells are essentially absent from IL-7Rα^−/−^ mice, in contrast to IL-7Rα^449F/449F^ mice. It is possible that insufficient IL-7Rα^−/−^ cells reached the T2 stage in the spleen, rendering them unable to fill the marginal zone niche, whereas sufficient IL-7Rα^449F/449F^ cells are present after the T2 stage to fill the marginal zone compartment. However, the T1-T2 defect was not intrinsic to the B cells themselves, as IL-7Rα^449F/449F^ T2 cells performed equally to WT T2 cells in chimeras, where both WT and IL-7Rα^449F/449F^ cells are present. We found that Notch proteins in T1 cells and levels of Notch-ligands in the spleen are normal, with increased levels of the Notch-regulator Deltex1 in IL-7Rα^449F/449F^ mice. Deltex1 was expressed at lower levels in TSLP-overexpressing mice [10], which had the opposite phenotype to IL-7Rα^449F/449F^ mice, with increased numbers of follicular B cells, suggesting that IL-7/TSLP may regulate deltex-1, and that deltex-1 may negatively regulate the level of follicular B cells in some manner.

We find it unlikely that defects in bone marrow B cell development in IL-7Rα^449F/449F^ mice (Unpublished data) is the reason behind the defective B cell development and survival in the spleen. IL-7Rα^449F/449F^ cells from mixed bone marrow chimeras show defective bone-marrow B cell development, but normal spleen T1, T2, follicular and marginal zone proportions. However, we cannot rule out that WT cells are having effects within the bone marrow to rescue spleen B cell development and survival.

Higher levels of IL-7 cannot rescue follicular B cell levels in IL-7Rα^449F/449F^ mice. This suggests that signals downstream of Tyr449 for follicular B cell development and survival are unique, and that enhanced signaling through motifs other than Y449 cannot compensate for the lack of signaling at Y449.

Levels of naïve antibody in IL-7Rα^449F/449F^ mice are similar to WT, with the notable exception of IgA. IL-7Rα^449F/449F^ mice, similar to IL-7Rα^−/−^ [24], lack Peyer's patches in the intestine (not shown). B cells within the Peyer's patches produce a large amount of IgA[25], so it seems possible that the absence of Peyer's patches in IL-7Rα^449F/449F^ mice is responsible for the lack of IgA. The related cytokines BAFF and APRIL are able to alter IgA levels in mice [26], [27]. Although BAFF levels are increased in IL-7Rα^449F/449F^ and IL-7Rα^−/−^, these mice have lower levels of IgA, suggesting that BAFF does not have a role in this process.

IL-7 signaling has a clear effect on splenic B cell development and in T cell biology [11]. However, the IL-7Rα^449F/449F^ mice had no defect in antibody responses to Influenza, T-dependent or T-independent antigens, suggesting that the cells which remain are capable of producing a functional antibody response. There was an exception to this however, in the defect in HES-specific response to *H. polygyrus*, which was dependent on IL-7Rα Tyr449 but not TSLP. It is unclear why IL-7Rα^449F/449F^ mice were unable to form normal antibody responses to *H. polygyrus*, and what is unique about the response to this pathogen that requires IL-7Rα Tyr449. It may represent a failure of Th2 T cell responses in IL-7Rα^449F/449F^ mice, or be symptomatic of a defective gut immune system.

We previously described a clear role for IL-7 (and not TSLP) and signaling downstream of IL-7Rα^449F/449F^ in the T cell response to Influenza A PR8 [17], but there was no difference in the primary antibody response to Influenza in IL-7Rα^449F/449F^ mice. However, this study primarily focused on effector CD4 and CD8 responses, not the ability of T cells to provide help to B cells, primarily mediated by T-follicular helper cells. IL-7Rα^449F/449F^ mice have similar numbers of CXCR5^+^ICOS^+^ T-follicular helper cells (not shown) which may allow normal T cell help from these cells.

IL-7 is a critical cytokine for the development of T and B cells that, in excess, allows the development of leukaemia in both mice and humans. We have shown that, despite regulating the survival of follicular B cells, blocking the IL-7Rα Tyr449 signaling does not prevent productive antibody responses. We have shown that the peripheral differences seen in IL-7Rα^449F/449F^ are due to defects within the spleen itself. IL-7 in the spleen controls the survival of splenic follicular B cells indirectly, via a mechanism that mostly likely relies on a RAG-dependent bone marrow derived cell. Coupled with the absence of any functional defect in the antibody responses to immunogens and infectious agents, this represents a clear target for potential therapies, combining the ability to block B cells cancers without any functional B cell immunodeficiency.

## Supporting Information

Table S1 Antibodies.
(PDF)
