# Correction: The Development and Survival but Not Function of Follicular B Cells Is Dependent on IL-7Rα Tyr449 Signaling

**DOI:** 10.1371/journal.pone.0093316

**Published:** 2014-03-19

**Authors:** The PLOS ONE Staff

**Notice of Republication**

This article was republished on March 4, 2014, to remove confidential or copyrighted material. Please download the article and supporting information again to view the corrected version.
