# Supplementary material for: The Development and Survival but Not Function of Follicular B Cells Is Dependent on IL-7Rα Tyr449 Signaling
Source: PLoS One. 2014 Feb 13;9(2):e88771. doi: 10.1371/journal.pone.0088771 (PMC3923819; doi:10.1371/journal.pone.0088771)
Supplement: Table S1 — Antibodies. (PDF) [file pone.0088771.s002.pdf]

Table S1 - Antibodies

Antibody	Label	Company	Clone
AA4.1	PE	eBioscience	AA4.1
B220	APC	eBioscience	RA3-6B2
B220	APC-Cy7	eBioscience	RA3-6B2
B220	APC-eFluor780	eBioscience	RA3-6B2
B220	FITC	eBioscience	RA3-6B3
BAFFR	APC	eBioscience	eBio7H22-E16
CD19	Alexa700	eBioscience	1D3
CD19	FITC	eBioscience	1D3
CD19	Pacific Blue	BioLegend	6D5
CD19	PE	BD Biosystems	1D3
CD21	PE	eBioscience	8D9
CD21	APC	eBioscience	8D9
CD23	PE-Cy7	eBioscience	B3B4
CD45.1	Biotin	BD Biosystems	A20
CD45.1	FITC	BD Biosystems	A20
CD45.1	PE	BD Biosystems	A20
CD45.2	APC	eBioscience	104
CD45.2	APC-Cy7	eBioscience	104
CD45.2	APC-eFluor780	eBioscience	104
IgM	PerCP-Cy5.5	eBioscience	II/41
IgM	PerCP-eFluor710	eBioscience	II/41
IgM	DyLight488	Jackson Immunoresearch	Polyclonal
IgD	V450	BD Bioscience	11-26c.2a
